# Social Interaction in Adolescent Rats with Neonatal Ethanol Exposure: Impact of Sex and CE-123, a Selective Dopamine Reuptake Inhibitor

**DOI:** 10.3390/ijms25021041

**Published:** 2024-01-15

**Authors:** Justyna Socha, Pawel Grochecki, Irena Smaga, Joanna Jastrzębska, Olga Wronikowska-Denysiuk, Marta Marszalek-Grabska, Tymoteusz Slowik, Robert Kotlinski, Małgorzata Filip, Gert Lubec, Jolanta H. Kotlinska

**Affiliations:** 1 Department of Pharmacology and Pharmacodynamics, Medical University of Lublin, Chodzki 4a, 20-093 Lublin, Poland; justynasocha97@gmail.com (J.S.); pawel.grochecki@umlub.pl (P.G.); 2 Department of Drug Addiction Pharmacology, Maj Institute of Pharmacology Polish Academy of Sciences, Smetna 12, 31-343 Krakow, Poland; smaga@if-pan.krakow.pl (I.S.); czyzyk@if-pan.krakow.pl (J.J.); mal.fil@if-pan.krakow.pl (M.F.); 3 Independent Laboratory of Behavioral Studies, Chair of Biomedical Sciences, Medical University of Lublin, Chodzki 4a, 20-093 Lublin, Poland; olga.wronikowska-denysiuk@umlub.pl; 4 Department of Experimental and Clinical Pharmacology, Medical University, Jaczewskiego 8b, 20-090 Lublin, Poland; marta.marszalek-grabska@umlub.pl; 5 Experimental Medicine Center, Medical University, Jaczewskiego 8, 20-090 Lublin, Poland; tymoteusz.slowik@umlub.pl; 6 Clinical Department of Cardiac Surgery, University of Rzeszow, 35-601 Rzeszow, Poland; robert.kotlinski@gmail.com; 7 Department of Neuroproteomics, Paracelsus Medical University, 5020 Salzburg, Austria; gert.lubec@meduniwien.ac.at

**Keywords:** adolescence, neonatal ethanol exposure, social behavior, selective dopamine reuptake inhibitor, CE-123

## Abstract

Children with fetal alcohol spectrum disorders (FASDs) demonstrate deficits in social functioning that contribute to early withdrawal from school and delinquency, as well as the development of anxiety and depression. Dopamine is involved in reward, motivation, and social behavior. Thus, we evaluated whether neonatal ethanol exposure (in an animal model of FASDs) has an impact on social recognition memory using the three-chamber social novelty discrimination test during early and middle adolescence in male and female rats, and whether the modafinil analog, the novel atypical dopamine reuptake inhibitor CE-123, can modify this effect. Our study shows that male and female rats neonatally exposed to ethanol exhibited sex- and age-dependent deficits in social novelty discrimination in early (male) and middle (female) adolescence. These deficits were specific to the social domain and not simply due to more general deficits in learning and memory because these animals did not exhibit changes in short-term recognition memory in the novel object recognition task. Furthermore, early-adolescent male rats that were neonatally exposed to ethanol did not show changes in the anxiety index but demonstrated an increase in locomotor activity. Chronic treatment with CE-123, however, prevented the appearance of these social deficits. In the hippocampus of adolescent rats, CE-123 increased BDNF and decreased its signal transduction TrkB receptor expression level in ethanol-exposed animals during development, suggesting an increase in neuroplasticity. Thus, selective dopamine reuptake inhibitors, such as CE-123, represent interesting drug candidates for the treatment of deficits in social behavior in adolescent individuals with FASDs.

## 1. Introduction

The term “fetal alcohol spectrum disorders” (FASDs) refers to a range of highly prevalent neurodevelopmental disorders resulting from prenatal alcohol exposure (PAE). Among the associated impairments across the entire spectrum, a broad range of behavioral problems classified as social in nature seems to be very characteristic [1,2,3]. PAE-related social behavior deficits emerge early in development and become more pronounced prior to and during adolescence, a critical period of development during which significant behavioral, cognitive, and physiological changes occur, including the onset of puberty, making adolescence a unique period of increased vulnerability to social behavior dysfunction. Importantly, the pervasive deficits in social functioning across the neurodevelopmental period have widespread implications for other domains, such as executive function and emotional processing [4,5].

In fact, in individuals with FASDs, the high rates of disruptive social behavior are frequently described in terms of impaired social competence, which can broadly be defined as effectiveness in social interaction or ability to employ social skills successfully within various interpersonal contexts [6,7]. Accordingly, adolescents and adults with FASDs have impaired socialization skills that include failure as a consequence of their own actions and lack of reciprocal friendships, as measured by the Vineland Adaptive Behavioral Scale, even when deficits in I.Q. are not evident [8]. Such impaired social behavior in individuals with FASDs may contribute to difficulties within the school environment, social rejection, trouble with the law, and later mental health problems [9].

In rodents, social behavior determines the establishment and maintenance of social structures and contains multiple components, including social affiliation, interaction, and recognition/discrimination [10]. In particular, social recognition, the ability to remember and discriminate individuals of the same species or even the social unit, is an essential and basic component of social behavior [11]. Data from animal models of PAE have shown neurobehavioral deficits parallel to those observed in individuals with FASDs, including disrupted play behavior and changes in social investigation [12,13].

The contribution of mesolimbic dopamine, the neurotransmitter involved in reward and motivation, has been particularly well-characterized in social play behavior [14]. Juvenile social play is one of the earliest forms of directed (non-mother) social behaviors that is rewarding and serves as a natural reinforcer. It is crucial for the development of behavioral flexibility, as well as the acquisition of social communication and cognitive competence, and may function to establish social organization and maintain cohesion in a group [15,16,17]. Thus, dopamine reuptake inhibitors or dopamine receptor agonists increase social play behavior [18] and the motivational salience of social play [19] (as seen in adolescent rats), whereas antagonism of either D1 or D2 dopamine receptors decreases social behavior [18]. It has been shown that dopamine neurons of the ventral tegmental area (VTA) are activated by conspecific interaction [20] and, moreover, this activity is also necessary for social novelty exploration [21]. Furthermore, alterations in synaptic properties of VTA dopamine neurons have been observed in autism mouse models [22], where they have been linked to impairments in maintaining social interest. In addition, published data show that FASDs are comorbid with autism spectrum disorders (ASD) and attention deficit hyperactivity disorder (ADHD) [23,24,25], where dysfunction of dopaminergic signaling leads to a series of developmental psychopathologies [26,27].

Indeed, published data show that prenatal and neonatal alcohol exposure disrupts the dopamine system, including reducing spontaneous activity of dopamine neurons, enhancing the response to dopamine agonists, and decreasing levels of the dopamine metabolite, homovanillic acid (HVA), in dopamine neurons [28,29,30]. For example, hyperactivity during early development can be induced through chemical depletion of midbrain dopamine neurons in neonatal rats [31,32] or by administering alcohol prenatally [33,34] or neonatally [35]. Moreover, the dopamine system is involved in executive and inhibitory control—areas in which FASDs children show marked deficits [36].

CE-123 is a low-affinity but highly selective dopamine transporter (DAT) inhibitor with good bioavailability [37]. This compound is a modafinil analog that inhibits dopamine reuptake with high specificity without causing an efflux of dopamine. CE-123 is a relatively weak (IC50 = 2.8 × 10^−6^ M [38]) but apparently very selective DAT inhibitor with respect to NET and SERT. Hence, it has a beneficial pharmacological profile and relatively low abuse liability [39]. Published data show that this modafinil analog increases extracellular dopamine in the nucleus accumbens (NAc).

In a laboratory setting, aspects of social functioning, such as social novelty discrimination/social recognition memory [40], can be observed and investigated using a three-chamber interaction behavior approach. Specifically, during this task, the test animals spend more time investigating a social stimulus and prefer a novel conspecific over a familiar one [41]. The current study assesses social novelty preference and recognition using a modified version of the three-chamber social test [42]. Herein, we evaluated, for the first time, whether chronic (10-day) administration of CE-123, a selective dopamine reuptake inhibitor, impacts social recognition deficits in animals with FASDs. Moreover, because the hippocampus is engaged in social learning, and the hippocampal dopamine endings affect learning and neuronal plasticity [43,44], we evaluated the impact of CE-123 on proteins involved in neuronal plasticity, such as the brain-derived neurotrophic factor (BDNF) and its receptor, the tropomyosin receptor kinase B (TrkB), as BDNF has been found in a previous study to mediate neuronal differentiation during development [45] and is needed for terminal differentiation of new neurons [46]. Our study aimed to determine whether neonatal ethanol exposure (a “third trimester exposure” model), a rat model for studying FASDs, induces social interaction deficits in early (postnatal day 28—PND28)- and middle (PND42)-adolescent animals [47], and whether these deficits are sex-dependent.

## 2. Results

### 2.1. Experiment 1: Effects of Neonatal Ethanol Administration on Social Novelty Discrimination in Adolescents

**Figure 1:**
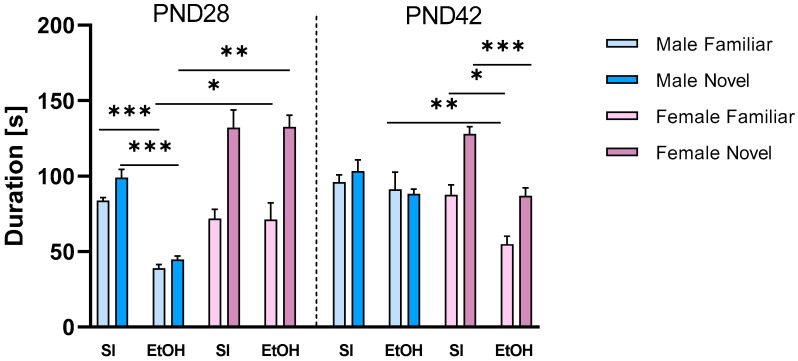
Effects of neonatal ethanol administration on social novelty discrimination in adolescents. * *p* < 0.05; ** *p* < 0.01; *** *p* < 0.001; n = 8–10/group. PND—postnatal day, SI—sham intubation (i.g.), EtOH—ethanol intubation (i.g.).

The three-way ANOVA of the results obtained in the discrimination phase of the social interaction test of rats at PND28 indicated the significant effect of ethanol [F (1, 70) = 26,19; *p* < 0.001], social stimulus (familiar or novel) [F (1, 70) = 54.05; *p* < 0.001], and sex factor [F (1, 70) = 53.15; *p* < 0.001]. Three-way ANOVA revealed the significant effect of social stimulus x sex [F (1, 70) = 26.19; *p* < 0.001] and ethanol x sex [F (1, 70) = 25.87; *p* < 0.001] interactions. Tukey’s post hoc test showed that ethanol treatment decreased the time spent in contact with both familiar (*p* < 0.001) and novel (*p* < 0.001) subjects in males. Such effects were not significant in females. Ethanol-treated females spent significantly more time both with familiar (*p* < 0.05) and novel (*p* < 0.001) counterparts compared to ethanol-treated males (Figure 1).

The three-way ANOVA of the results obtained in the discrimination phase of the social interaction test of rats in PND42 indicated the significant effect of ethanol [F (1, 72) = 25.13; *p* < 0.001] and social stimulus (familiar or novel) [F (1, 72) = 16.98; *p* < 0.001], with a lack of effect of sex factor [F (1, 72) = 1.341; *p* > 0.05]. Three-way ANOVA revealed the significant effect of social stimulus x sex [F (1, 72) = 13.36; *p* < 0.001] and ethanol x sex [F (1, 70) = 8.304; *p* < 0.05] interactions. Tukey’s post hoc test showed that ethanol treatment decreased the time spent in contact with both familiar (*p* < 0.05) and novel (*p* < 0.001) counterparts in females. Moreover, ethanol-treated female rats spent significantly less time with familiar counterparts compared to ethanol-treated males (*p* < 0.01). There were no significant differences between ethanol-treated males and females in time spent investigating novel subjects (*p* > 0.05) (Figure 1).

### 2.2. Experiment 2a: Effect of CE-123 Administration on Neonatal Ethanol-Induced Deficits in Social Novelty Discrimination in Adolescents

**Figure 2:**
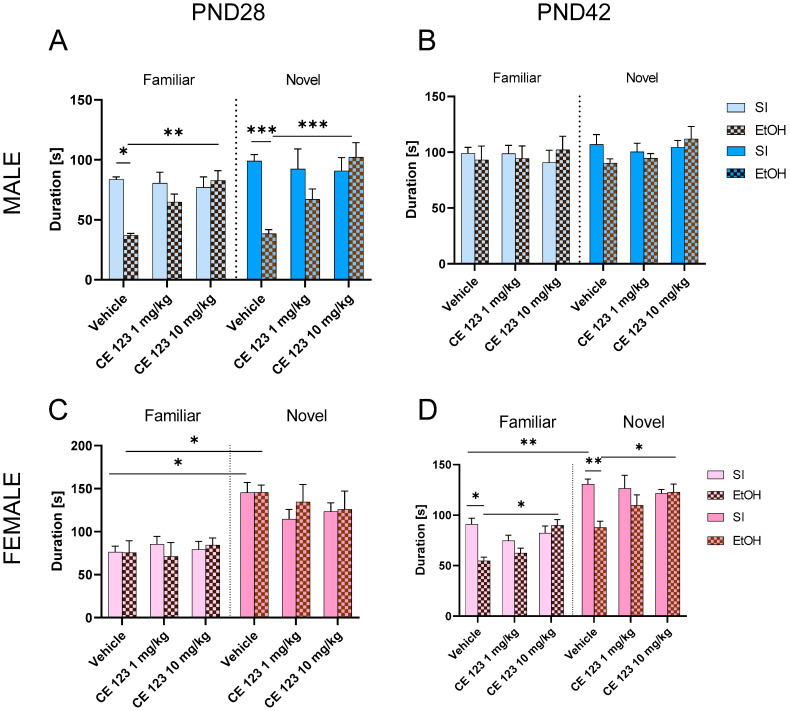
Effect of CE-123 administration on neonatal ethanol-induced deficits in social novelty discrimination in (**A**) PND28 male; (**B**) PND42; (**C**) PND28 female; and (**D**) PND42 female rats. * *p* < 0.05; ** *p* < 0.01; *** *p* < 0.001; n = 10/group. PND—postnatal day, SI—sham intubation (i.g.), EtOH—ethanol intubation (i.g.).

The three-way ANOVA of the results obtained in the discrimination phase of the social interaction test of males at PND28 indicated a significant effect of ethanol [F (1, 112) = 16.34; *p* < 0.001], CE-123 administration [F (2, 112) = 6.32; *p* < 0.01], and ethanol x CE123 interaction [F (2, 112) = 10.93; *p* < 0.001]. Tukey’s post hoc test showed that ethanol treatment decreased the time spent with familiar (*p* < 0.05) and novel (*p* < 0.001) subjects. CE-123 administration at the dose of 10 mg/kg reversed this effect with familiar (*p* < 0.01) and novel (*p* < 0.001) subjects, respectively (Figure 2A).

The three-way ANOVA of the results obtained in the discrimination phase of the social interaction test of males at PND42 did not show any significant effect of ethanol F (1, 112) = 0.1925; *p* > 0.05], CE-123 administration [F (2, 112) = 0.4335; *p* > 0.05], or social stimulus (familiar or novel) [F (1, 112) = 0.4335; *p* > 0.05] (Figure 2B).

The three-way ANOVA of the results obtained in the discrimination phase of the social interaction test of females at PND28 revealed a significant effect of social stimulus (familiar or novel) [F (1, 106) = 49.58; *p* < 0.001] but did not show any significant effect of ethanol F (1, 106) = 0.0792; *p* > 0.05] or CE-123 administration [F (2, 106) = 0.5637; *p* > 0.05]. Tukey’s post hoc test showed that both control (*p* < 0.05) and ethanol-treated (*p* < 0.05) females preferred novel subjects at PND28. There was no significant effect of CE-123 on this behavior (Figure 2C).

In contrast, three-way ANOVA analysis of results obtained at PND42 showed the significant effect of ethanol [F (1, 108) = 16.46; *p* < 0.001], CE-123 administration [F (2, 108) = 3.925], social stimulus (familiar or novel) [F (1, 108) = 100.5] factors, and ethanol x CE-123 interaction [F (2, 108) = 9.89; *p* < 0.001]. The post hoc test showed that control females preferred novel subjects at PND42 (*p* < 0.01). Ethanol administration decreased the time spent with familiar subjects (*p* < 0.05), but CE-123 administration at the dose of 10 mg/kg reversed this effect (*p* < 0.05). Moreover, ethanol administration decreased the time spent with novel objects (*p* < 0.01), and CE-123 at the dose of 10 mg/kg reversed this deficit (Figure 2D).

#### 2.2.1. Experiment 2b: Effects of Neonatal Ethanol Administration on Locomotor Activity in Adolescents: Effect of CE-123

Two-way analysis of the results obtained in the locomotor activity test of PND28 males revealed the significant effect of ethanol administration [F (1, 28) = 15.61; *p* > 0.001]. However, the analysis did not show a significant effect of CE-123 administration [F (2, 28) = 3.290; *p* > 0.05] or interaction of the factors [F (2, 28) = 2.131; *p* > 0.05]. Tukey’s post hoc test indicated a significant difference between control and ethanol-treated groups (*p* < 0.01). Moreover, the post hoc test revealed significant difference between ethanol-treated and ethanol/CE-123-treated rats at the dose of 10 mg (*p* < 0.05).

In early-adolescent females, these factors did not affect the locomotor activity, with the following results: ethanol [F (1, 31) = 1.893; *p* > 0.05], CE-123 administration [F (2, 31) = 0.1515; *p* > 0.05], and interaction [F (2, 31) = 0.5633; *p* > 0.05].

In mid-adolescent rats, ethanol administration did not affect animal behavior in males [F (1, 33) = 0.1126; *p* > 0.05] or females [F (1, 34) = 0.0675; *p* > 0.05]. Similarly, CE-123 had no influence on locomotor activity in males [F (2, 33) = 0.3073; *p* > 0.05] or females [F (2, 34) = 0.6991). The interaction of these factors was not significant (Table 1).

#### 2.2.2. Experiment 2c: Effects of Neonatal Ethanol Administration on Anxiety Behavior in Adolescents: Effect of CE-123

In early-adolescent males (PND28), the two-way ANOVA of the results obtained in the EPM calculated as the anxiety index did not reveal any significant effect of ethanol [F (1, 31) = 0.2333; *p* > 0.05], CE-123 administration [F (2, 31) = 0.4286; *p* > 0.05], or interaction of these factors [F (2, 31) = 0.1646; *p* > 0.05]. Similarly, in PND28 females, ethanol [F (1, 31) = 0.0031; *p* > 0.05], CE-123 administration [F (2, 31) = 4.434; *p* > 0.05], and interaction of these factors [F (2, 31) = 0.8943; *p* > 0.05] did not affect the behavior of the tested animals.

Furthermore, the two-way ANOVA analysis of the results obtained in mid-adolescent males (PND42) did not show any significant influence of ethanol [F (1, 32) = 0.1053; *p* > 0.05], CE-123 administration [F (2, 32) = 0.3959; *p* > 0.05], or interaction of these factors [F (2, 32) = 0.2576; *p* > 0.05]. In PND42 females, ethanol [F (1, 33) = 3.556; *p* > 0.05], CE-123 administration [F (2, 33) = 0.6018; *p* > 0.05], and interaction of these factors [F (2, 31) = F (2, 33) = 0.9998] did not affect the behavior of the tested animals. Tukey’s post hoc tests did not show any significant differences between tested groups in the EPM test (Table 1).

#### 2.2.3. Experiment 2c: Effects of Neonatal Ethanol Administration on BDNF and TrkB Expression in the Hippocampus of Adolescent Rats: Effect of CE-123

**Figure 3:**
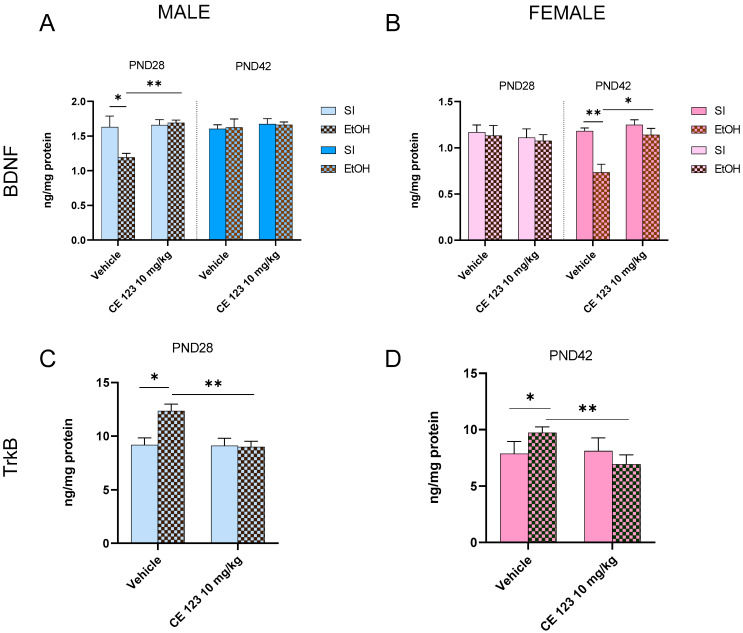
Effects of neonatal ethanol administration on BDNF expression in PND28 and PND42 (**A**) male and (**B**) female rats, as well as on TrkB expression in (**C**) PND28 male and (**D**) PND42 female rats in the hippocampus. Effect of CE-123. * *p* < 0.05; ** *p* < 0.01; n = 10/group. PND—postnatal day, SI—sham intubation (i.g.), EtOH—ethanol intubation (i.g.).

The three-way ANOVA of the results obtained in ELISA assays indicated the significant effect of CE-123 administration [F (1, 33) = 6.160; *p* < 0.05] on BDNF expression in the hippocampus of male rats. However, other factors, e.g., ethanol administration [F (1, 33) = 2.504; *p* > 0.05], the age of rats [F (1, 33) = 2.419; *p* < 0.05], and their interactions, were not significant. Tukey’s post hoc test showed significant differences between ethanol-treated and control males at PND28 (*p* < 0.05), but CE-123 administration reversed this effect (*p* < 0.01). This effect was not seen at PND42 (Figure 3A).

In female rats, the three-way ANOVA indicated the significant effect of ethanol administration [F (1, 33) = 8.268; *p* < 0.01]. Moreover, the three-way ANOVA revealed the significant effect of CE-123 administration x age of rats [F (1, 32) = 7.316; *p* < 0.05] and ethanol x age of rats [F (1, 33) = 4.994; *p* < 0.05]. Tukey’s post hoc test showed significant differences between ethanol-treated and control females at PND42 (*p* < 0.01); CE-123 administration reversed this effect (*p* < 0.05) (Figure 3B).

The two-way ANOVA of the results obtained in the ELISA assay of TrkB indicated the significant effect of ethanol [F (1, 16) = 5.624; *p* < 0.05], CE-123 administration [F (1, 16) = 5.624; *p* < 0.05], and the interaction of these factors F (1, 16) = 6.482; *p* <0.05] in male early-adolescent rats. Tukey’s post hoc test showed significant differences between ethanol-treated and control males (*p* < 0.05); CE-123 administration reversed this effect (*p* < 0.01) (Figure 3C).

In mid-adolescent female rats, the two-way ANOVA showed the significant effect of CE-123 administration [F (1, 16) = 9.460; *p* < 0.01] and ethanol x CE-123 interaction F (1, 16) = 13.09; *p* < 0.01] on TrkB expression in the hippocampus. Tukey’s post hoc test showed significant differences between ethanol-treated and control females (*p* < 0.05), and CE-123 administration reversed this effect (*p* < 0.01) (Figure 3D).

### 2.3. Experiment 3: Effect of Neonatal Ethanol Administration on Short-Term Memory in NOR. Effect of CE-123

**Figure 4:**
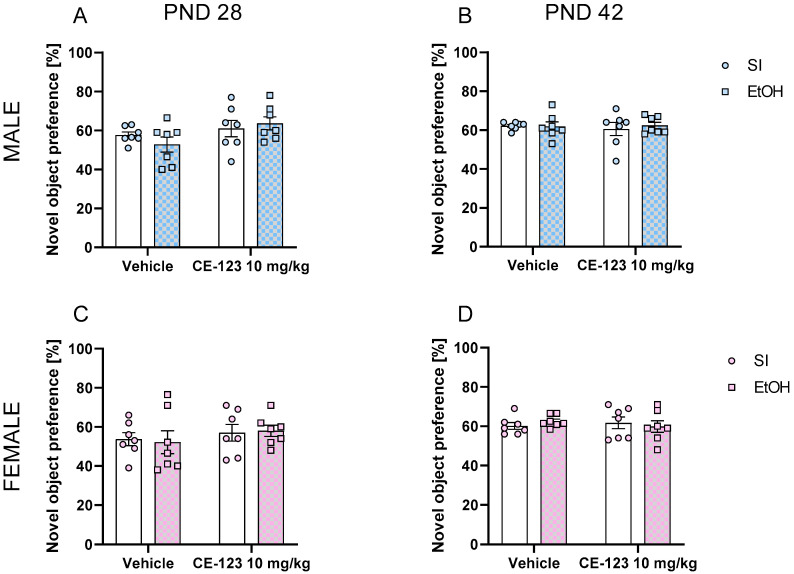
Effect of neonatal ethanol administration on short-term memory in (**A**) PND28 male, (**B**) PND42, (**C**) PND28 female, and (**D**) PND42 female rats; n = 6–7/group. PND—postnatal day, SI—sham intubation (i.g.), EtOH—ethanol intubation (i.g.) in NOR task. Effect of CE-123.

In male early-adolescent rats (PND28), the two-way ANOVA of the results obtained in NOR revealed the significant effect of CE-123 administration [F (1, 24) = 4.285; *p* < 0.05] but no significant effect of ethanol [F (1, 24) = 0.0983; *p* > 0.05] or interaction of these factors [F (1, 24) = 1.228; *p* > 0.05]. Tukey’s post hoc test did not indicate any differences between tested groups (Figure 4A).

In male mid-adolescent rats (PND42), the two-way ANOVA did not reveal any significance of CE-123 [F (1, 24) = 0.2286; *p* > 0.05] or ethanol [F (1, 24) = 0.1229; *p* > 0.05] administration. Moreover, Tukey’s post hoc test did not indicate any differences between tested groups (Figure 4B).

In females, the two way-ANOVA of the results obtained in NOR did not reveal any significant effect of CE-123 in early-adolescent (PND28) [F (1, 24) = 1.150; *p* > 0.05] or mid-adolescent [F (1, 24) = 0.0571; *p* > 0.05] rats. Similarly, ethanol exposure had no significant effect in early-adolescent (PND28) [F (1, 24) = 0.0041; *p* > 0.05] or mid-adolescent [F (1, 24) = 0.0135; *p* > 0.05] female rats. Tukey’s post hoc test did not indicate any differences between tested groups (Figure 4C,D).

## 3. Discussion

Our study shows that male and female rats subjected to ethanol in the neonatal period (a rat model of FASDs) exhibited sex- and age-dependent deficits in social novelty discrimination in early (male) and middle (female) adolescence. Importantly, we demonstrated that these deficits are specific to the social domain and not simply due to more general deficits in learning and memory. This is because both sexes of neonatal ethanol-exposed rats did not exhibit changes in short-term recognition memory in the NOR task. Furthermore, these rats did not show changes in the anxiety index; however, an increase in locomotor activity was observed in early adolescence in males. Chronic treatment with CE-123, a DAT inhibitor, prevented the appearance of the social deficits associated with neonatal ethanol exposure. In the hippocampus of adolescent rats, CE-123 increased BDNF and decreased its signal transduction TrkB receptor expression level in ethanol-treated animals during development, suggesting an increase in neuroplasticity.

For many mammalian species, adolescence is a critical developmental period during which social interactions are prominent and help shape proper brain development [48]. However, prenatal ethanol exposure disturbs social investigation [13], including social interactions [49], and this effect is dependent on the timing, duration, and amount of ethanol exposure. In our study, we performed the social novelty discrimination test (using a standard three-chambered apparatus) that assesses the ability of rodents to discriminate between a previously investigated familiar from a novel adolescent coefficient [50]. Our data showed that females with developed FASDs model were like controls in ability and preference in discriminating between novel and familiar social stimuli at an early age of testing (PND28), but deficits in social novelty discrimination were observed in mid-adolescence (PND42). Thus, this finding generally confirms other studies showing that adolescent (PND42) [49,51] or adult [51] females prenatally exposed to ethanol demonstrate social avoidance.

Stress and anxiety-like behavior can have an impact on social interaction. Earlier findings have shown that the coefficient of social preference/avoidance is an index of anxiety-like behavior in the social context because this measure is extremely sensitive to both anxiogenic [52,53] and anxiolytic [53,54] manipulations. Specifically, when tested in an unfamiliar, anxiety-provoking environment, animals demonstrate social avoidance regardless of age and sex. In contrast, testing under familiar, non-stressful circumstances results in social preference [53]. In published studies, experimental subjects are socially isolated for at least several days [53] or hours [55] prior to testing, whereas our animals were housed with their same-sex littermates and were socially deprived for only 30 min prior to testing. Deprivation from social interactions is stressful, particularly for younger animals [56], and the reported sex-related differences in social behavior may reflect differential sensitivity to such a stressor between males and females [57]. Furthermore, prenatal or neonatal ethanol exposure can induce anxiety-like behavior in adolescence [58,59]. Published data show [60,61] that animals with prenatal ethanol exposure reveal anxiety-like behavior that is dependent on the test employed. Thus, although our findings demonstrated that neonatally ethanol-treated animals did not show changes in anxiety-like behavior in the EPM test, the decrease in social investigation and/or social preference displayed by ethanol-exposed females on PND42 may reflect anxiety-like alterations evident under social circumstances.

In turn, our study of male rats showed a deficit in social novelty discrimination and preference in early (PND28) but not middle (PND42) adolescence. However, changes in locomotor activity of animals could have an impact on the outcome of the social novelty discrimination test. Indeed, our results show that neonatal ethanol administration increased locomotion in early-adolescent males, but such results were not observed in mid-adolescent females that also indicated deficits in social interaction. Thus, we hypothesized that social interaction deficits were not the result of changes in locomotor activity. Consequently, our study confirmed previously existing studies’ findings [42,51,60,62,63] that the social consequences of prenatal/neonatal ethanol exposure differ based on age and sex.

In human beings, adolescence can be associated with high levels of novelty- and sensation-seeking behavior [64,65,66], and males are reported to engage in more sensation-seeking behavior than females across all age categories [66]. In rodents, males display a higher preference for the novel object than females at mid-adolescence (PND40), with no sex difference at early adolescence [67]. Interestingly, our data showed that female Wistar rats exhibit greater levels of social behavior related to males, which is in contrast to a previous study [68]. Nevertheless, reports of sex-related differences in social behavior appear to be in part strain-dependent [53,69,70]; therefore, female Wistar rats might display a higher preference for novelty than males.

Social behavior deficits following PAE have often been described as a secondary effect of general cognitive deficits in learning and memory [71]. Used in our study, the social novelty discrimination test assesses the ability of rodents to discriminate between a previously investigated familiar from a novel adolescent rat [50], thus offering a direct measure of social recognition memory in a complex social context [72]. Therefore, utilization of the NOR task with parameters identical to those of social novelty discrimination testing allowed us to examine whether ethanol exposure in the neonatal period induced deficits in adolescent rats in recognition memory and whether the deficits were associated with a general impairment in learning and memory. Although ethanol-treated male rats showed a tendency toward reduced recognition of new objects in early adolescence, the results were not significant. Thus, the current finding confirmed our findings [73] and others’ [74,75,76] findings that neonatal/prenatal ethanol exposure does not induce deficits in short-term recognition memory in either sex of adolescent rats. Importantly, our findings indicate that there was no relationship between social recognition and object recognition memory.

The social novelty discrimination test is thought to be a test of social recognition in a separate cognitive domain from the visual learning and memory involved in NOR [77]. Social novelty discrimination involves evaluation and responses to social cues, which involve the accessory olfactory bulb and the neuropeptides oxytocin and vasopressin, which enhance acquisition and consolidation, respectively [11], and none of which are involved in NOR. Indeed, oxytocin knockout mice show specific deficits in social novelty discrimination without any alteration in non-social memory [77,78], confirming that social novelty discrimination and NOR map to different cognitive domains. In addition, dopamine is thought to contribute to the effects of oxytocin and vasopressin on social processes [79]. Dopaminergic neurotransmission has been linked to various cognitive processes, including spatial memory, object recognition memory and social memory [80,81,82]. Recent research shows that social interaction itself is a highly rewarding/motivating experience that engages the dopaminergic reward circuitry, perhaps especially during adolescence [83]. Thus, the novel atypical dopamine transporter inhibitor, CE-123 (a modafinil analog with an improved pharmacodynamics profile) [38,55,84,85,86], has been recently shown to protect social recognition memory deficits [39,55,86]. Among other behavioral effects, CE-123 did not induce hyperlocomotion or anxiogenic or stereotypic behavior in young rats [37]. In a prior study, intraperitoneal administration of CE-123 into Sprague–Dawley rats improved working memory in the radial maze [87] and enhanced cognitive flexibility without triggering unnecessary impulsive responding [38]. Furthermore, our previous research showed that CE-123 improved memory acquisition and memory retrieval in the spatial memory task and ameliorated ADHD-like activity in animals with FASDs [35].

The present data extend our studies concerning the precognitive effects of CE-123 in a rat model of FASDs. So far, we have shown that a single administration of CE-123, a novel DAT inhibitor, before testing attenuated locomotor hyperactivity (an ADHD-like symptom) in adolescent rats and reversed learning disabilities in adult rats exposed to ethanol over the neonatal period [35]. In our current study, CE-123 has been shown to protect against social recognition memory deficits in the novelty discrimination test in adolescent animals when given chronically over 10 days before the test. The results were successful in the adolescent male and female rats. We hypothesize that CE-123 can have an impact on the acquisition of memory and stabilize/strengthen the engram during acquisition for social recognition against interference in rats. The effect was dose-dependent, and the CE-123 dose of 10 mg/kg was the most effective. Furthermore, CE-123 alone did not have an impact on the behavior of control animals, but it reversed ethanol-induced hyperlocomotion in early-adolescent male rats. Thus, our present study suggests that this modafinil analog may represent an interesting drug candidate for FASDs therapy in humans.

We previously reported that neonatal ethanol exposure (PND4-9) induced changes in dopamine (D1, D2, and D5) receptor mRNA expression in the striatum, hippocampus, and prefrontal cortex of adult rats [35]. In that study, CE-123 increased dopamine levels in different brain regions [55], and the published data indicate that infusion of a D1 agonist into the frontal cortex or nucleus accumbens may improve short-term social recognition in rats [88]. Other data show that projections from an ensemble of CA1 neurons (preferentially reactivated in the presence of a previously encountered conspecific compared with an unfamiliar conspecific) to the nucleus accumbens are necessary for social discrimination [89]. The hippocampus and striatum (including nucleus accumbens) are areas in which increased dopamine signaling might contribute to a “stabilization” of the social recognition memory trace, thereby making it resistant against interference [90,91].

Overall, our study shows that chronic CE-123 administration reversed social recognition deficits in a rat model of FASDs. It is known that dopamine regulates the long-term potentiation (LTP) and that BDNF is required for the induction and maintenance of dopamine-induced LTP [92]. Consequently, adolescent rats with developed FASDs exhibit decreased BDNF expression in the hippocampus, and CE-123 treatment returns this expression to the control group; this finding correlates with behavioral outcomes.

### Conclusions and Indications

Our findings show for the first time (a) neonatal ethanol exposure (model of FASDs)-induced deficits in short-term social recognition, but not object recognition memory, in adolescent rats of both sexes; (b) that chronic administration of CE-123, a selective DAT inhibitor, prevented social recognition memory deficits in animals with FASDs; and (c) that in these animals, CE-123 modified the expression of proteins involved in neuronal plasticity in the hippocampus, such as BDNF and its receptor. Thus, our study describes the crucial role of dopamine in social recognition memory deficits in animals with FASDs. However, one limitation of the current study is that we are not able to directly assess the interrelationship between physiological biochemistry and psychology of behavior, which appears to be determined by the influence of dopamine. Another limitation is that we did not determine the impact of other receptors and neurotransmitters (such as acetylcholine) or environmental factors (pharmaceutical xenobiotics) on social behavior in the rat model of FASDs. Thus, although modafinil analogs, such as CE-123, represent interesting drug candidates for the treatment of deficits in social behavior in adolescent individuals with FASDs, further study will reveal whether the results in animals can be transferred to humans.

## 4. Material and Methods

### 4.1. Animals

The experiments were carried out according to the National Institute of Health Guide for the Care and Use of Laboratory Animals and the European Community Council Directive for Care and Use of Laboratory Animals (86/609/EEC). In addition, they were approved by the Local Ethics Committee (25/2023). We used Wistar rats that were bred and housed in the vivarium of the Medical University of Lublin, Poland. They were maintained on a 12 h/12 h light/dark cycle, with lights on at 8:00 a.m., and at a controlled temperature of 22 ± 1 °C and 55 ± 10% humidity. The animals had free access to food and water. For breeding, one female and one male rat were housed for one week together. Following 3 weeks post-mating, the females were assessed for parturition each morning and evening. After this, on PND3, pups were assigned to experimental groups of equal numbers of males and females. On PND21, animals were weaned and housed by sex with littermates, with 2–3 animals per cage. Behavioral tests began at PND28 and PND42 using three separate animal cohorts. All experiments were conducted between 8:00 a.m. and 4:00 p.m.

### 4.2. Drugs and Neonatal Treatment

On PND3, male and female rat pups were assigned to 2 treatment groups that were used in the behavioral experiments: sham-intubated (SI) (male/female) and ethanol-intubated (male/female) (FASDs model). Ethanol (95% *w*/*v*, POCH BASIC, Avantor Performance Materials Poland S.A., Gliwice, Poland) was given on PND4-9 according to the method described by Goodfellow et al. [93] and MacIlvane et al. [94] (as a “3rd trimester exposure” model). Pups received ethanol at a dose of 5.0 g/kg/day, 22, 66% *v*/*v*, delivered via intragastric intubation (i.g.) in milk (Bebilon 1 Pronutra Plus) solution. This dose of ethanol produces significant neurotoxicity during the third trimester equivalent and may lead to neurobehavioral deficits [95]. On PND21, the animals were separated (weaned) from their mothers.

(*S*)-CE-123 (5-((benzhydrylsulfinyl)methyl)thiazole) (CE-123), a modafinil analogue, was synthesized in the Lubec Laboratory (University of Vienna, Austria). On each administration day (PND10-20), CE-123 was freshly dissolved in a vehicle containing 1% DMSO (10%) and 3.3% Tween 80 (15%) diluted in 0.9% NaCl (75%). The administration procedure and doses of CE-123 were selected based on previous studies [35,38,40].

### 4.3. Procedures

#### 4.3.1. Three-Chamber Social Novelty Discrimination Test

The three-chamber test was performed to measure the social approach and social preference in male and female rats during early (PND28) and middle (PND42) adolescence. In brief, one day before testing (habituation phase), experimental animals were placed alone in the testing apparatus for 30 min to make them more familiar with the testing apparatus, because social interactions are notably higher when animals are tested in a familiar but not an unfamiliar chamber [96,97]. The testing apparatus was a rectangular three-chamber box, with two lateral chambers (30 × 35 × 35 cm) connected to a central chamber (15 × 35 × 35 cm) (Ugo Basile, Gemonio, Italy). Each lateral chamber contained a small Plexiglass cylindrical cage. On the test day, each experimental rat was marked on its side with indelible ink and then placed alone in a holding cage for 30 min [98,99]. The animal was then placed into the testing apparatus for 5 min. Following this acclimatization period, the tested rat was briefly confined to the central chamber, while a non-manipulated rat (Stranger 1, i.e., one cage mate) confined in a small wire cage was randomly placed in one of the outer chambers. An identical empty wire cage was placed in the other chamber. The test animal was then allowed to explore the arena for a further 5 min. The social interaction test in rats measures social investigation. This is usually defined as the time spent in each chamber and/or the time spent in proximity to distinct wire cages. However, as time spent in each chamber does not necessitate active and direct investigation of the stimulus, we evaluated only one variable—the time in proximity to the distinct wire cages.

To investigate the preference for social novelty, a novel unfamiliar rat (Stranger 2, i.e., the offspring of a non-treated, non-familiar rat) was then placed in the empty cage, and the test animal was allowed to explore the arena for a further 5 min separated by a 30 min retention period [72]. The unfamiliar, introduced rats were always age- and sex-matched animals that had not been socially isolated before testing and were unfamiliar with both the test apparatus and the experimental animals with which they were paired for testing [53,100]. Time spent engaging in investigatory behavior with the novel, unfamiliar rat was recorded to assess preference for social novelty. The arena was cleaned between animals with 15% ethanol and dried with a paper towel.

#### 4.3.2. Elevated Plus-Maze (EPM) Test

The EPM test utilizes a black-painted, wooden apparatus composed of four crossed arms forming a plus sign raised 50 cm above the laboratory floor, with a central platform placed at the intersection of the arms. Two arms of the maze are open (50 × 10 cm), and two are closed 50 × 10 × 40 cm). The EPM test was performed in accordance with our previous study [96,101] in a quiet, dark room, and the central square of the maze was illuminated uniformly with low-intensity, constant 15 W (136 lm) lighting located 80 cm above the maze. The entire experiment was recorded using a video camera and computer software (ANY-maze video tracking system 6.3, Stoelting Co. Wood Dale, IL, USA). Observation of animal behavior in the experiment included: (1) measuring the time that the rat stays in the arms of the EPM; and (2) measuring the number of entries to the arms of the EPM.

The anxiety index was calculated using the formula: 1 − [(time in open arms/total test time) + (entries in open arms/all entries)]/2 [101]. The EPM test was performed during early adolescence (PND28) and in late adolescence (PND42) after the habituation phase of the social novelty discrimination test.

#### 4.3.3. Locomotor Activity

Locomotor activity was measured in all rats in specially designed cages made of transparent plastic (locomotor activity boxes) connected to a computer. The infrared sensors in these boxes, positioned 45 and 100 mm above the floor, measured the activity of the animals. The cages were placed in a soundproof room with constant lighting (40 W–300–500 lm). Locomotor activity was assessed as the distance traveled by the animal in meters (m). Horizontal activity (as the distance traveled) of the animals was measured for 15 min after placing the animals in the apparatus. Locomotor activity testing was performed during PND28 and during PND42, after the habituation phase of the social novelty discrimination test and right after the EPM test.

#### 4.3.4. Novel Object Recognition (NOR)

For all animals (male and female; PND28/42), this NOR task was carried out in a Plexiglass box (40 × 40 × 40 cm) illuminated with ~20 lux light in a quiet room. Animals were placed in the apparatus for 30 min before every session of the NOR task. The procedure [102,103] included 3 sessions, i.e., (1) habituation, followed by the next day; (2) a training session (5 min); and (3) a testing session (5 min) with a 30 min interval. The animals did not receive any injections during the training/testing session.

During the training session, two identical objects were placed in diagonal corners of the box. During the testing session, one of the objects was replaced by a novel object different in color and shape. Each animal was separately placed in the center of the box facing one of the remaining empty corners. Both the training and the testing sessions were recorded to provide further analysis of animal behaviors. Object recognition was manually scored by a blind experimenter and calculated as the percentage value. The set of objects was chosen based on preliminary studies that showed no innate preference between selected objects. After each session of the NOR task, the animals returned to their home cages. During the training and testing sessions, the total distance travelled (cm) was calculated using EthoVision XT V 15 (Noldus, Wageningen, Netherlands).

#### 4.3.5. Enzyme-Linked Immunosorbent Assay (ELISA)

The protein levels were validated using a Rat-Brain-Derived Neurotrophic Factor ELISA Kit (E0476Ra; Bioassay Technology Laboratory, Shanghai, China) and a Rat Tyrosine Kinase B ELISA Kit (E1598Ra; Bioassay Technology Laboratory, Shanghai, China) following the manufacturers’ protocols. Briefly, HIP was homogenized in cold PBS (pH 7.4) with cocktails of protease and phosphatase inhibitors (Sigma-Aldrich, St. Louis, MI, USA) and centrifuged for 5 min at 5000× *g*. A bicinchoninic acid assay (BCA) protein assay kit (Serva, Heidelberg, Germany) was used for protein concentration measurement in the supernates. The absorbance of duplicates of each sample and the standards were measured at a wavelength of λ = 450 nm using a Multiskan Spectrum spectrophotometer (Thermo LabSystems, Philadelphia, PA, USA). The concentration of proteins was calculated from standard curves and expressed as ng/mg of protein.

### 4.4. Experimental Design

Three sets of experiments were conducted.

#### 4.4.1. Experiment 1. Effects of Neonatal Ethanol Administration on Social Novelty Discrimination in Adolescents

Male/female SI- or ethanol-treated rats were separated from their mothers (PND21) and assigned to experimental groups based on (1) sex (males and females), (2) FASDs/SI model, and (3) age of rats (early and middle adolescence). Next, the animals (PND28/PND42) were subjected to a three-chamber social novelty discrimination test according to the method described above.

#### 4.4.2. Experiment 2. Effect of CE-123 Administration on Neonatal Ethanol-Induced Deficits in Social Novelty Discrimination in Adolescents

Male/female SI- or ethanol-treated rats (PND21) were assigned to experimental groups based on (1) sex (males and females), (2) FASDs/SI model, (3) administered substance (vehicle/CE-123), and (4) age of rats (early and middle adolescence). CE-123 was administered intraperitoneally (i.p.) at the dose of 1 and 10 mg/kg (at a rate of 1 mL/kg body weight) for 10 days beginning from PND10, once a day, similarly to other cognitive enhancers [104]. Animals in the control group were administered the vehicle alone. The three-chamber social novelty discrimination test was carried out similarly to Experiment 1. The rats were subjected to locomotor activity and EPM tests. After completion of the behavioral procedures, the animals were sacrificed, and the hippocampus was dissected, snap-frozen, and stored at −80 °C. Next, BDNF and TrkB were measured using ELISA.

#### 4.4.3. Experiment 3. Effects of Neonatal Ethanol Administration on Adolescent Rat Behavior in NOR task. Effect of CE-123

Animals that were SI- or ethanol-treated were subjected to experimental groups based on (1) sex, (2) FASDs/SI model, (3) administered substance (vehicle/CE-123), and (4) age of rats. CE-123 was administered as it was in Experiment 2 at the dose of 10 mg/kg. The NOR task was performed according to the method described above.

### 4.5. Statistical Analysis

The obtained results were analyzed using Prism v. 8.0.0 for Windows (GraphPad Software, San Diego, CA, USA). The statistical significance of effects from behavioral and molecular tests was analyzed by applying two- or three-way analysis of variance (ANOVA) with repeated measures. This was followed by Tukey’s post hoc test. The results were presented as means ± standard errors of means (SEM) of values, where a *p* value less than 0.05 was considered statistically significant for all tests.

## Figures and Tables

**Table 1:** Effects of neonatal ethanol administration on locomotor activity and anxiety behavior in adolescents. Effect of CE-123. Results are presented as mean ± standard errors (SEM). ** *p* < 0.01 vs. SI/vehicle; ^#^ *p* < 0.05 vs. ethanol/vehicle.

Group	Sex	PND28		PND42	
		Distance Traveled (cm) ±SEM	Anxiety Index ±SEM	Distance Traveled (cm) ±SEM	Anxiety Index ±SEM
**Ethanol/Vehicle**	**Male**	3262.91 ± 92.29 **	0.768 ± 0.036	4100.13 ± 496.03	0.850 ± 0.043
	**Female**	2919.55 ± 294.53	0.708 ± 0.024	4484.85 ± 452.90	0.872 ± 0.045
**Ethanol/CE5**	**Male**	2906.025 ± 339.32	0.755 ± 0.028	4623.93 ± 798.43	0.837 ± 0.035
	**Female**	2796.35 ± 593.49	0.816 ± 0.037	3810.84 ± 322.08	0.820 ± 0.049
**Ethanol/CE10**	**Male**	2297.05 ± 138.817 ^#^	0.720 ± 0.021	3738.01 ± 525.92	0.847 ± 0.028
	**Female**	3147.12 ± 160.23	0.860 ± 0.032	4497.07 ± 508.13	0.825 ± 0.027
**SI/Vehicle**	**Male**	1997.09 ± 23.77	0.757 ± 0.049	3591.81 ± 669.02	0.825 ± 0.052
	**Female**	2392.80 ± 375.03	0.762 ± 0.033	3332.97 ± 540.27	0.762 ± 0.033
**SI/CE5**	**Male**	2312.40 ± 264.21	0.792 ± 0.066	4072.04 ± 801.28	0.807 ± 0.041
	**Female**	2828.65 ± 182.51	0.793 ± 0.040	4481.99 ± 855.82	0.745 ± 0.033
**SI/CE10**	**Male**	1941.97 ± 291.66	0.747 ± 0.046	4271.73 ± 519.40	0.869 ± 0.045
	**Female**	2493.66 ± 204.98	0.824 ± 0.051	4622.22 ± 399.80	0.824 ± 0.051

## Data Availability

The data presented in this study are available upon request from the corresponding author.
